# MRI-based quantification of intratumoral heterogeneity for predicting recurrence risk in ER+/HER2− breast cancer

**DOI:** 10.1186/s13244-026-02342-0

**Published:** 2026-06-27

**Authors:** Yang Chen, Jie Shi, Jing Chen, Lizhi Xie, Jin Ye, Wei Tang, Qin Xiao, Yan Huang, Yajia Gu, Weijun Peng

**Affiliations:** 1 Department of Radiology, Fudan University Shanghai Cancer Center, Shanghai, China; 2 Department of Oncology, Shanghai Medical College, Fudan University, Shanghai, China; 3 MR Research China, GE Healthcare, Beijing, China; 4 Department of Ultrasound, Secondary Sanatorium of Air Force Healthcare Center for Special Services, Hangzhou, China; 5 Department of Data Science & AI, Faculty of Information Technology, Monash University, Melbourne, VIC Australia

**Keywords:** Breast neoplasms, Neoplasm recurrence, Magnetic resonance imaging, Radiomics, Artificial intelligence

## Abstract

**Objectives:**

The need for a cost-effective, rapid, and increasingly accessible alternative to the 21-gene assay prompted this study, which developed a novel MRI-based intratumoral heterogeneity score (ITHscore) to quantify tumor heterogeneity and integrated it with radiomic and clinical features to predict the 21-gene recurrence score (RS).

**Materials and methods:**

This retrospective study included ER+/HER2− breast cancer patients who underwent 21-gene assay and preoperative MRI at our institution (April 2017–March 2019). Patients were randomly split into training (70%) and internal test (30%) cohorts, with an external test cohort from the public Duke-Breast-Cancer-MRI dataset. Tumor volumes of interest were automatically segmented using a pre-trained Scalable and Transferable U-Net framework, followed by k-means clustering to compute the ITHscore. Predictive models for the RS were built using clinical, radiomics, and ITHscore features with the support vector machine method, and evaluated by receiver operating characteristic curves.

**Results:**

The institutional dataset comprised 452 patients (training: 316 (187 high-risk, 129 low-risk); internal test: 136 (80 high-risk, 56 low-risk)), while the external Duke cohort included 230 patients (44 high-risk, 186 low-risk). The ITHscore was significantly elevated in high-risk patients (*p* < 0.001), and its incorporation into the clinical-radiomic model improved RS prediction, yielding AUCs of 0.86 for the internal test cohort and 0.82 for the external test cohort.

**Conclusions:**

In this exploratory study, the ITHscore, which held promise as a noninvasive and intuitive means of characterizing intratumoral heterogeneity, demonstrated a potential incremental value for predicting RS in patients with ER+/HER2− breast cancer.

**Critical relevance statement:**

The MRI-derived quantification of intratumoral heterogeneity facilitates simple and quantitative assessment of tumor heterogeneity and demonstrates potential incremental value in providing a rapid, cost-effective, and accessible prediction of the recurrence score in ER+/HER2− breast cancer.

**Key Points:**

- There is a need for a cost-effective, rapid, and increasingly accessible alternative to the 21-gene assay for predicting recurrence risk in ER+/HER2− breast cancer.
- The intratumoral heterogeneity score demonstrated potential as a noninvasive, intuitive, and quantitative biomarker for characterizing intratumoral heterogeneity and predicting recurrence score.

**Graphical Abstract:**

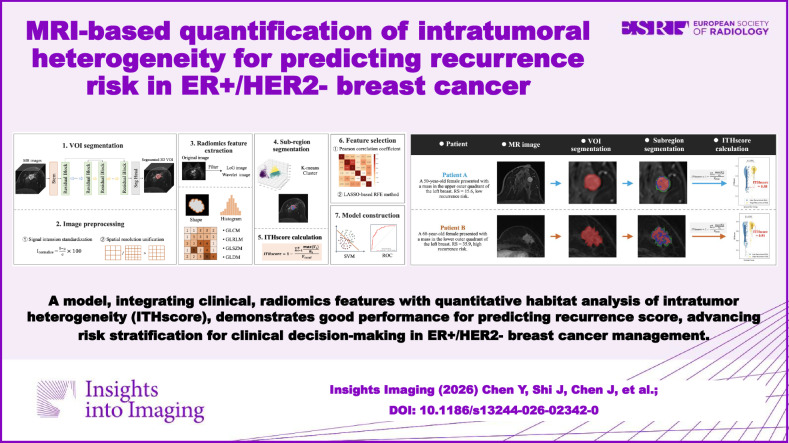

## Introduction

Breast cancer exhibits significant heterogeneity [1], wherein patients with identical molecular subtypes and comparable clinical stages may demonstrate divergent prognostic outcomes [2]. Oncotype DX (Genomic Health) 21-gene assay detects the expression of 21 specific genes of the lesion and employs a proprietary algorithm to generate a recurrence score (RS) [3], which reflects intra-tumor heterogeneity (ITH) to some extent. This score serves as a critical clinical tool, predicting 10-year postoperative recurrence risk and guiding adjuvant chemotherapy decisions in early-stage estrogen receptor (ER)-positive, human epidermal growth factor receptor 2 (HER2)-negative breast cancer [4, 5]. However, the assay is associated with high cost, prolonged turnaround time, and technical complexity [6, 7], underscoring the clinical need for a cost-effective, rapid, and convenient biomarker as an alternative to the 21-gene assay RS.

Radiomics enables the extraction of high-dimensional imaging features imperceptible to visual assessment, capturing global tumor characteristics and ITH through hundreds to thousands of quantifiable parameters [8–10]. However, radiomic analysis is grounded in the assumption of uniform distribution of ITH, which means that the extracted features are averages of all pixel characteristics—a global perspective that could not fully capture inter-regional differences within the tumor [9, 10]. The recently emerged habitat analysis offers a local perspective by mapping distinct subregions (“habitats”) to visualize spatial heterogeneity and tumor microenvironment interactions [11]. Both radiomics and habitat analysis showed promise in prognostic prediction [12–15]. Acknowledging the complementary prognostic value of both approaches, our recent work introduced the intratumoral heterogeneity score (ITHscore)—a novel, noninvasive quantitative index derived from MRI that comprehensively characterizes tumor heterogeneity by integrating both global and local features.

The RS, radiomics, and habitat analysis all converge on the concept of tumor heterogeneity, with each providing valuable prognostic insights. This common biological basis suggests a probable correlation among them. Initial investigations into radiomics for RS prediction have been reported, yet these efforts are often hampered by limited sample sizes, single-center designs [6, 7], and the use of non-contemporary RS cutoffs [13, 16]. Moreover, to our knowledge, no published research has investigated the potential of habitat analysis to predict RS.

Therefore, our study leveraged a dual-center cohort from our institution and a public database with a twofold strategy: first, to develop a novel ITHscore for intuitively quantifying intratumoral heterogeneity; and second, to construct a predictive model for the RS by synthesizing radiomic features, the ITHscore, and clinical characteristics, and to investigate its utility as a cost-effective, rapid, and increasingly accessible approach for prognostic stratification and adjuvant chemotherapy guidance in ER+/HER2− breast cancer.

## Materials and methods

### Patients

**Fig. 1:**
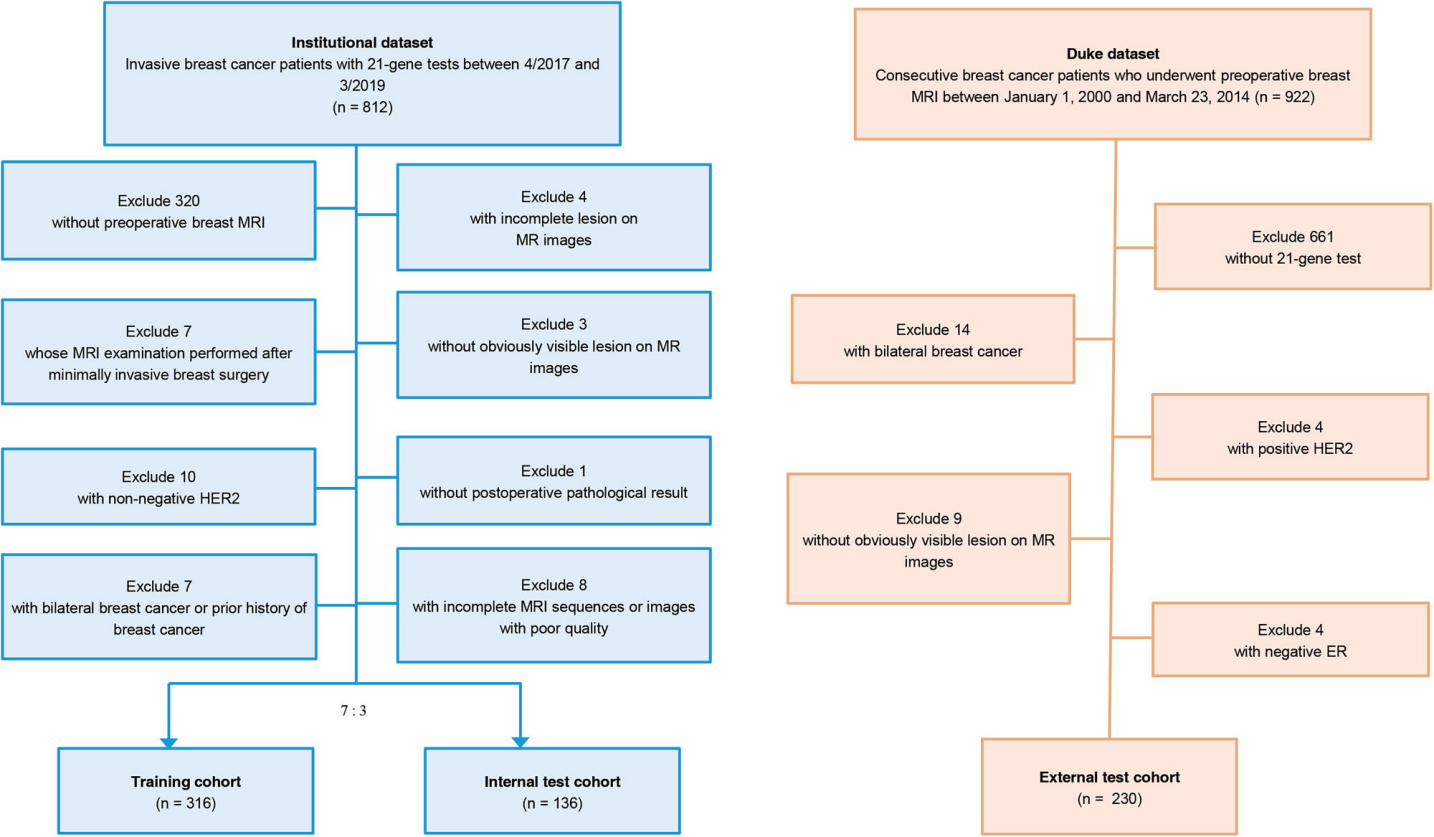
Patient selection in the Institutional and Duke datasets. ER, estrogen receptor; HER2, human epidermal growth factor receptor 2

This retrospective study received approval from the institutional review board of our hospital (1612167-18) and was granted a waiver of informed consent. The study cohorts comprised patients from two distinct centers (Fig. 1):

1. Institutional dataset: Patients who underwent 21-gene test and preoperative breast MRI at our institution between April 2017 and March 2019 were included. All patients subsequently underwent definitive breast cancer surgery at our hospital, with available surgical pathology reports. Pathological characteristics, including histological type, progesterone receptor status, tumor size, lymph node status, and invasive malignancy grade, were extracted from the final surgical pathology reports.
2. Duke dataset: This retrospective study utilized data from a publicly accessible database [17] (freely available at: https://wiki.cancerimagingarchive.net/plugins/servlet/mobile?contentId=70226903#content/view/70226903, accessed May 3, 2022). The dataset comprised 1150 consecutive breast cancer patients who underwent preoperative breast MRI between January 1, 2000, and March 23, 2014. Patients who had ER+/HER2− breast cancer and underwent 21-gene test were selected for further analysis.

Exclusion criteria: bilateral breast cancer, prior history of breast cancer, HER2−positive or HER2−equivocal status, ER-negative status, receipt of minimally invasive surgical intervention prior to MRI, MRI-nonvisible or incompletely visualized lesions, incomplete MRI sequences, images with poor quality due to significant artifacts or excessive noise, and postoperative pathological result unavailability.

### Oncotype DX 21-gene assay

All enrolled patients underwent the Oncotype DX 21-gene assay, which quantifies expression levels of 16 cancer-related genes and 5 reference genes to generate an RS ranging from 0 to 100. The RS provides prognostic information, with higher scores indicating both increased 10-year distant recurrence risk and greater benefit from adjuvant chemotherapy. In accordance with the National Comprehensive Cancer Network clinical practice guidelines for breast cancer (available at NCCN.org, Version 2.2026), patients were stratified into two risk categories based on a validated RS cutoff of 26: high-risk recurrence (RS ≥ 26) and low-risk recurrence (RS < 26).

### MRI scanning protocol

All study participants of the institutional dataset underwent breast MRI examinations using one of four clinical MRI scanners, with detailed technical specifications provided in Table S1. Breast MRI was performed with patients in the standard prone position, with both breasts appropriately positioned within a dedicated 16-channel breast coil to ensure optimal image quality. Our dynamic contrast-enhanced (DCE) MRI protocol included an initial non-enhanced acquisition followed by three to five post-contrast dynamic phases. The first contrast-enhanced series was initiated 30–46 s following contrast administration, with two to four subsequent acquisitions performed consecutively without temporal gaps. Each dynamic phase was completed within 38–40 s. For contrast enhancement, we utilized Magnevist (Bayer HealthCare Pharmaceuticals Inc.) administered intravenously at a standard dose of 0.1 mmol/kg of body weight. The contrast agent was delivered via power injector at a rate of 1.5–2.0 mL/s, followed by a 20 mL saline flush at the same injection rate to ensure complete delivery of the contrast bolus.

The external test cohort from the Duke dataset underwent prone-position breast MRI examinations utilizing ten distinct scanner systems. As detailed in Table S1, all acquisitions were obtained in the transverse plane, with the imaging protocol consisting of DCE sequences that incorporated one unenhanced phase followed by four post-contrast phases. The median acquisition time between successive contrast-enhanced phases was 131 s. Regarding contrast administration, different gadolinium-based contrast agents were employed: gadobutrol (Gadavist, Bayer Healthcare) was administered to one patient (0.4%), gadopentetate dimeglumine (Magnevist, Bayer Healthcare) to 128 patients (55.7%), and gadobenate dimeglumine (Multihance, Bracco) to 72 patients (31.3%). In 29 cases (12.6%), the specific contrast agent information was not available. All contrast administrations followed a standardized weight-based protocol of 0.2 mL/kg.

### Volume of interest (VOI) segmentation

**Fig. 2:**
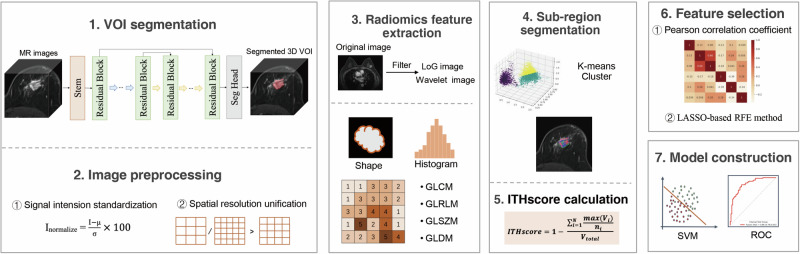
Framework of this study. VOI, volume of interest; LoG, Laplacian of Gaussian; GLCM, gray-level co-occurrence matrix; GLRLM, gray-level run-length matrix; GLSZM, gray-level size zone matrix; GLDM, gray-level dependence matrix; LASSO, least absolute shrinkage and selection operator; RFE, recursive feature elimination

A flow chart of the study design is provided in Fig. 2. Tumoral VOIs were automatically segmented from the last phase of DCE (CL) 3D medical images using a pre-trained Scalable and Transferable U-Net (STU-Net) framework [18]. The segmentation outputs were visually inspected by two radiologists with 7 and 20 years of experience who had no access to RS results, to ensure anatomical plausibility, and minor corrections were made if necessary. In approximately 20% of cases, automatic segmentation outputs underwent minor manual correction. The magnitude of these corrections was quantitatively small: the median Dice similarity coefficient between automatic and manually corrected contours was 0.96 (interquartile range: 0.93–0.98), and these adjustments were predominantly limited to subtle boundary refinements rather than substantial modifications of the overall tumor extent. To assess the potential impact of segmentation variability, an additional segmentation robustness experiment was conducted using 30 randomly selected cases. For each case, ITHscore was extracted using both the automatically generated VOI and the manually corrected VOI. The agreement between the two ITHscore measurements was high, with an intraclass correlation coefficient of 0.87. The model code for automatic segmentation was described in the open-source platform (https://github.com/uni-medical/STU-Net).

### Radiomics feature extraction

Given the variability in MRI acquisition parameters across different scanners used in this study, standardized image preprocessing was performed prior to feature extraction. First, signal intensity normalization was applied using the following transformation:

$${{{{\rm{I}}}}}_{{{{\rm{normalize}}}}}=\frac{I-\mu}\sigma\times100$$

where I_normalize_ represents the standardized image signal intensity, I denotes the original image signal intensity, and μ and σ correspond to the mean and standard deviation of I, respectively. Subsequently, all DCE images were resampled to a uniform spatial resolution of 0.703125 × 0.703125 × 1.48148 mm to ensure dimensional consistency across the datasets. These systematic approaches enable robust characterization of intratumoral heterogeneity while accounting for technical variations in multicenter imaging data acquired from different scanner platforms.

Comprehensive radiomic feature extraction was performed using PyRadiomics (version 1.3.1, PyRadiomics Community). A total of 1046 quantitative imaging features were extracted from each tumoral VOI, comprising 14 shape features, 18 first-order statistical features and 68 texture features, 100 of which were extracted from the original image, 258 from Laplacian of Gaussian-filtered images (applied with δ = 2, 3, and 4), and 688 from wavelet-filtered images.

### Subregion segmentation and ITHscore calculation

To capture the ITH at the subregional level, we performed subregion segmentation within each tumor VOI. The VOI mask was binarized to extract the voxel intensities from the tumor region. The extracted voxel values were standardized using z-score normalization and then subjected to unsupervised clustering. Specifically, a k-means clustering algorithm was applied with the number of clusters set to three, thereby partitioning the tumor into three spatially distinct subregions based on intensity similarity.

To quantify spatial ITH, an ITHscore was calculated from the subregion masks. Voxels with the same cluster label were grouped into spatially contiguous regions (connected components). The number and size of connected components were used to evaluate the degree of spatial fragmentation, which contributes to the ITHscore calculation. For each tumor, these subregion-level results were aggregated to derive a 3D ITHscore, where higher values indicate more fragmented and spatially diverse patterns of heterogeneity. The ITHscore was defined as:

$${ITHscore}=1-\frac{{\sum }_{i=1}^{N}\frac{\max ({V}_{i})}{{n}_{i}}}{{V}_{{total}}}$$

where represents the set of connected components in cluster, denotes the largest component volume in cluster, is the number of connected components, and is the total tumor volume.

### Feature selection and radiomics signature construction

Prior to model development, all continuous features in the training cohort underwent z-score normalization. The same normalization parameters (mean and standard deviation derived from the training cohort) were subsequently applied to both internal and external test cohorts to maintain consistency. To mitigate dimensionality and reduce overfitting, we implemented a two-stage feature selection approach combining Pearson correlation analysis and recursive feature elimination (RFE) using only the training cohort data. In the first stage, pairwise Pearson correlation coefficients were computed for all feature combinations. Feature pairs demonstrating high collinearity (Pearson coefficient > 0.9) were identified, and within each correlated pair, the feature with higher average correlation coefficients was systematically eliminated while retaining the lower correlation feature. For the second stage, RFE was implemented using the least absolute shrinkage and selection operator as the base estimator. The number of selected features ranged from 1 to 10% of the training cohort sample size, with incremental addition of one feature at each iteration.

To address class imbalance between high-risk and low-risk recurrence groups in the training cohort, we applied the synthetic minority oversampling technique (SMOTE) prior to model training, while SMOTE was not applied to either the internal or external test cohorts. The prediction model was developed using linear support vector machine (SVM) methodology, and model performance was quantitatively assessed through receiver operating characteristic (ROC) curve analysis. The classification threshold was determined using the Youden index from the ROC curve based on the training cohort, which maximizes the sum of sensitivity and specificity. The same fixed threshold was then applied to the internal and external cohorts to evaluate model performance. Bootstrap resampling was used to estimate the variability of model performance and to generate confidence intervals for the area under the ROC curve (AUC) values. Shapley Additive exPlanations (SHAP) analysis was performed to interpret model output, and calibration assessment was applied to assess the model’s predictive capabilities.

### Statistical analysis

Continuous variables were summarized as mean ± standard deviation or median (with the first and third quartiles), whereas categorical variables were reported as frequencies and percentages. Intergroup comparisons were performed as follows: normally distributed continuous variables (assessed via Kolmogorov-Smirnov test and homogeneity of variance) were compared using the independent Student’s *t*-test, while non-normally distributed continuous variables were analyzed using the Mann–Whitney U test. Categorical variables were compared using the chi-square test or Fisher’s exact test, as appropriate. All statistical analyses were conducted using SPSS (Version 25.0), while radiomics analysis was performed in Python (Version 3.8) and RStudio (Version 1.4.1103). A two-tailed *p*-value < 0.05 was considered statistically significant.

## Results

### Patient characteristics

The training cohort comprised 316 patients, including 187 classified as high-risk and 129 as low-risk. The internal test cohort consisted of 136 patients (80 high-risk, 56 low-risk), while the external test cohort included 230 patients (44 high-risk, 186 low-risk) (Fig. 1). The clinical characteristics of all enrolled patients are summarized in Table 1.

**Table 1:** Characteristics of patients

Characteristics	Training cohort (*n* = 316)			Internal test cohort (*n* = 136)			External test cohort (*n* = 230)		
	Low recurrence risk (*n* = 129)	High recurrence risk (*n* = 187)	*p*-value	Low recurrence risk (*n* = 56)	High recurrence risk (*n* = 80)	*p*-value	Low recurrence risk (*n* = 186)	High recurrence risk (*n* = 44)	*p*-value
Age	51.7 ± 9.3*	51.5 ± 9.9*	0.825	52.7 ± 9.1*	53.2 ± 8.1*	0.714	55.3 ± 9.7*	55.1 ± 11.3*	0.965
Postmenopausal	53 (41.1)	99 (52.9)	**0.038**	33 (58.9)	44 (55.0)	0.649	109 (58.6)	29 (65.9)	0.374
Pathology	129	187	0.949	56	80	0.922	186	44	0.347
Invasive ductal carcinoma	117 (90.7)	170 (90.9)		50 (89.3)	71 (88.8)		117 (62.9)	31 (70.5)	
The other invasive breast carcinoma	12 (9.3)	17 (9.1)		6 (10.7)	9 (11.3)		69 (37.1)	13 (29.5)	
Progesterone receptor positive	123 (95.3)	164 (87.7)	**0.021**	56 (100)	67 (83.8)	**0.002**	175 (94.1)	34 (77.3)	**0.001**
Tumor size	129	187	0.840	56	80	0.697	186	44	**0.039**
pTI	89 (69.0)	127 (67.9)		36 (64.3)	54 (67.5)		104 (55.9)	17 (38.6)	
pTII/III	40 (31.0)	60 (32.1)		20 (35.7)	26 (32.5)		82 (44.1)	27 (61.4)	
Negative lymph node	116 (89.9)	160 (85.6)	0.252	47 (83.9)	72 (90.0)	0.292	151 (81.2)	39 (88.6)	0.241
Invasive malignancy grade	124	177	**0.001**	54	77	0.112	142	32	**< 0.001**
Grade I	5 (4.0)	11 (6.2)		1 (1.9)	1 (1.3)		38 (26.8)	4 (12.5)	
Grade II	111 (89.5)	128 (72.3)		50 (92.6)	63 (81.8)		94 (66.2)	16 (50.0)	
Grade III	8 (6.5)	38 (21.5)		3 (5.6)	13 (16.9)		10 (7.0)	12 (37.5)	

Bold values mean statistically significant

* Data are means ± standard deviation

A significant association was observed between progesterone receptor (PR) status and the RS across all cohorts. The proportion of PR-positive tumors was markedly higher in the low-recurrence-risk group compared to the high-risk group in the training, internal test, and external test cohorts. In both the training and external test cohorts, invasive malignancy grade III was significantly more prevalent in patients with high-risk than low-risk. However, this distribution did not reach statistical significance in the internal test cohort. Furthermore, the training cohort demonstrated a significant enrichment of postmenopausal women at the high-risk. In the external test cohort, high-risk patients presented with larger invasive tumor sizes. No other clinical characteristics demonstrated significant differences between the high- and low-recurrence-risk patients.

### ITHscore

**Fig. 3:**
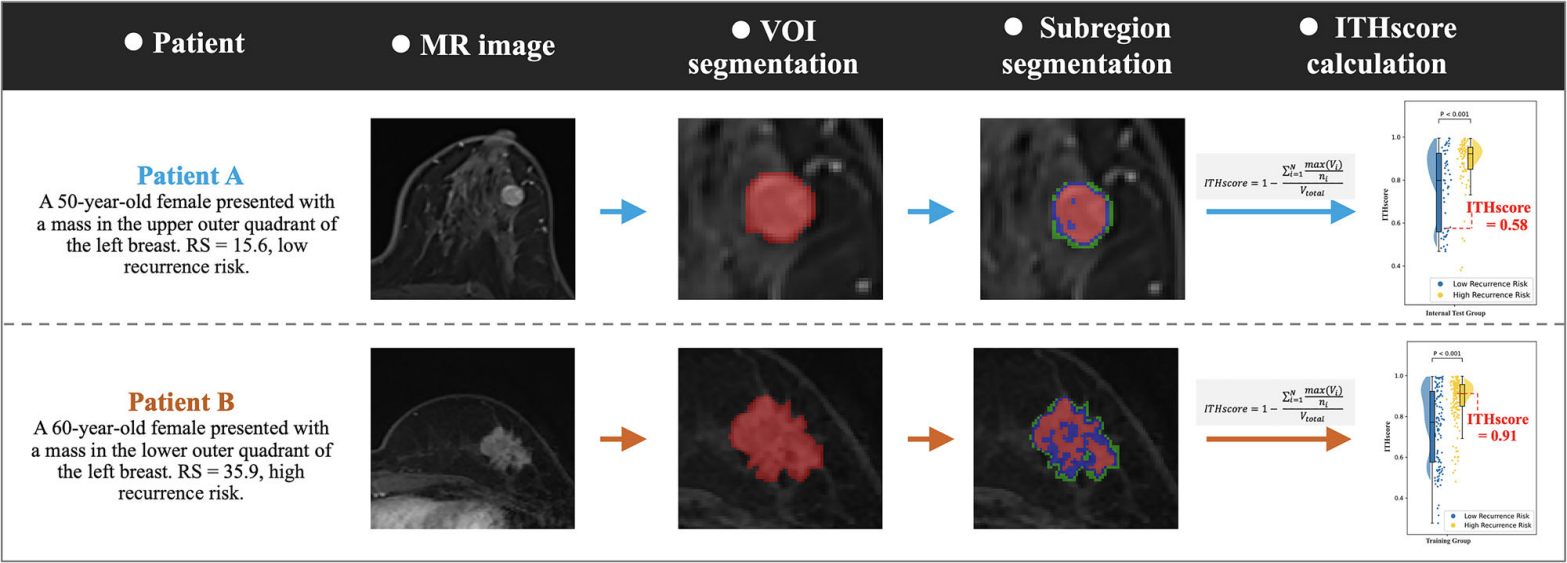
Example implementation of the habitat clustering process and intratumoral heterogeneity score (ITHscore) generation in two patients with ER+/HER2− breast cancer. VOI, volume of interest

Across the training, internal test, and external test cohorts, the median ITHscore was significantly higher in the high-risk patients compared to the low-risk patients (*p* < 0.001, Fig. S1). The median values for the low-risk versus high-risk groups were 0.772 (0.576–0.924) vs. 0.912 (0.850–0.957) in the training cohort, 0.798 (0.558–0.925) vs. 0.922 (0.850–0.953) in the internal test cohort, and 0.832 (0.728–0.966) vs. 0.965 (0.931–0.983) in the external test cohort, respectively. Figure 3 illustrates the habitat clustering process and ITHscore calculation in two representative patients.

### Performance of prediction models

**Fig. 4:**
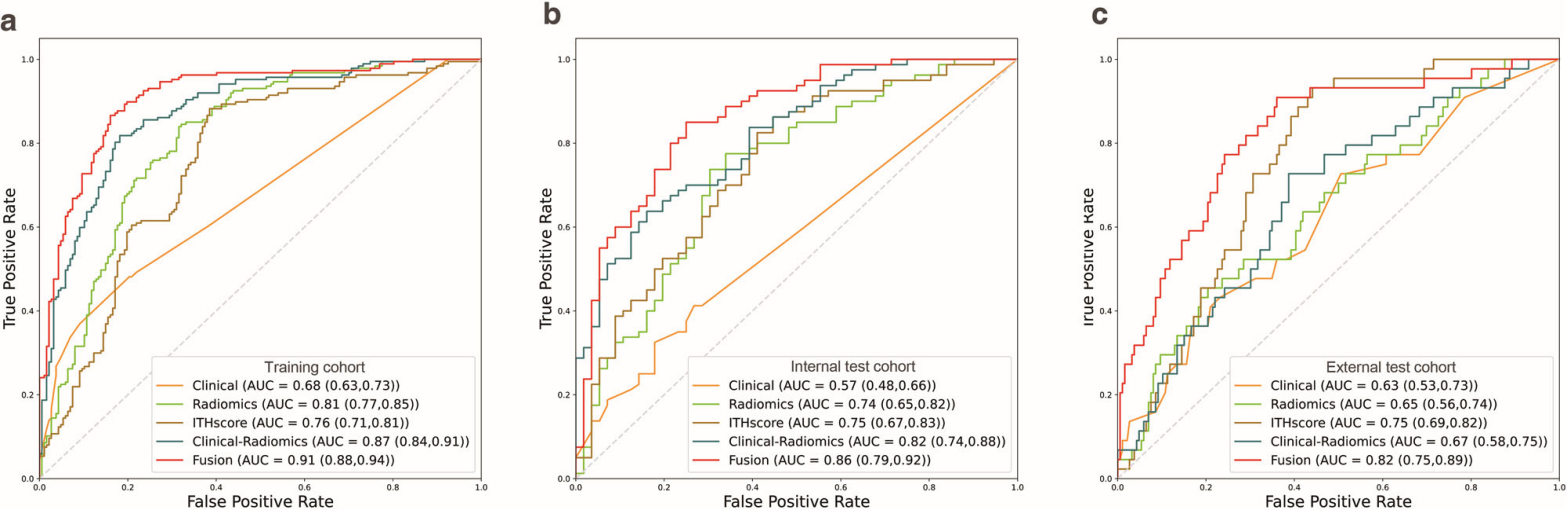
Receiver operating characteristic (ROC) curves of five different models for differentiating between low (RS < 26) and high recurrence risk (RS ≥ 26) in ER+/HER2− breast cancer patients in the training (**a**), internal test (**b**), and external test (**c**) groups. AUC, area under the ROC curve

**Fig. 5:**
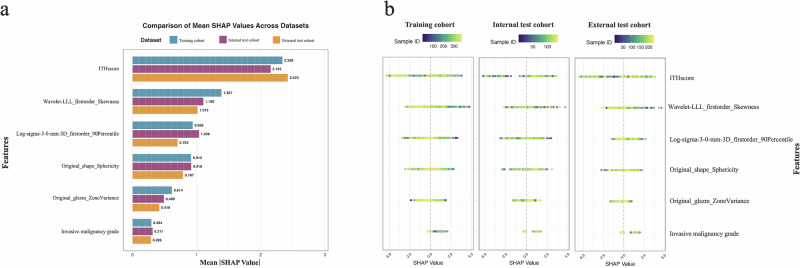
Feature importance and Shapley Additive exPlanations (SHAP) value visualization of the fusion model. Bar plots of mean absolute SHAP values (**a**) ranking the six features by their contribution to model prediction in the training, internal test, and external test cohorts. The intratumoral heterogeneity score (ITHscore) is identified as the most important predictor. Heatmap plots (**b**) showing SHAP values of the six selected features for individual patients across the three cohorts

We constructed five distinct prediction models based on clinical features, conventional radiomic features, and the ITHscore (Fig. 4, Table 2); the specific features included in each model are detailed in Table S2. The predictive model utilizing the ITHscore alone achieved AUC values of 0.76, 0.75, and 0.75 in the training, internal test, and external test cohorts, respectively. Notably, integrating the ITHscore into the clinical-radiomics model enhanced its performance, yielding a fusion model with improved AUCs of 0.91, 0.86, and 0.82 across the three respective cohorts. A Pearson correlation heatmap of the features within the fusion model is provided in Fig. S2. SHAP analysis demonstrated that the ITHscore was the most important predictor among the six features incorporated in the fusion model, consistently ranking first in the training, internal, and external test sets (Fig. 5). The calibration curves indicated the excellent accuracy of the fusion model (Fig. S4).

**Table 2:** Performance of different models

Model	Training cohort		Internal test cohort		External test cohort	
	AUC (95% CI)	ACC/SEN/SPE	AUC (95% CI)	ACC/SEN/SPE	AUC (95% CI)	ACC/SEN/SPE
Fusion	0.91 (0.88–0.94)	0.84/0.88/0.81	0.86 (0.79–0.92)	0.76/0.71/0.82	0.82 (0.75–0.89)	0.50/0.93/0.39
Clinical-Radiomics	0.87 (0.84–0.91)	0.79/0.78/0.81	0.82 (0.74–0.88)	0.66/0.65/0.68	0.67 (0.58–0.75)	0.53/0.82/0.46
ITHscore	0.76 (0.71–0.81)	0.74/0.86/0.62	0.75 (0.67–0.83)	0.73/0.85/0.55	0.75 (0.69–0.82)	0.55/0.95/0.45
Radiomics	0.81 (0.77–0.85)	0.75/0.81/0.68	0.74 (0.65–0.82)	0.68/0.66/0.71	0.65 (0.56–0.74)	0.36/0.91/0.23
Clinical	0.68 (0.63–0.73)	0.64/0.48/0.79	0.57 (0.48–0.66)	0.53/0.38/0.75	0.63 (0.53–0.73)	0.47/0.77/0.39

*ITHscore* intratumoral heterogeneity score, *AUC* area under the receiver operating characteristic curve, *CI* confidence interval, *ACC* accuracy, *SEN* sensitivity, *SPE* specificity

### Model performance in patient subgroups

**Fig. 6:**
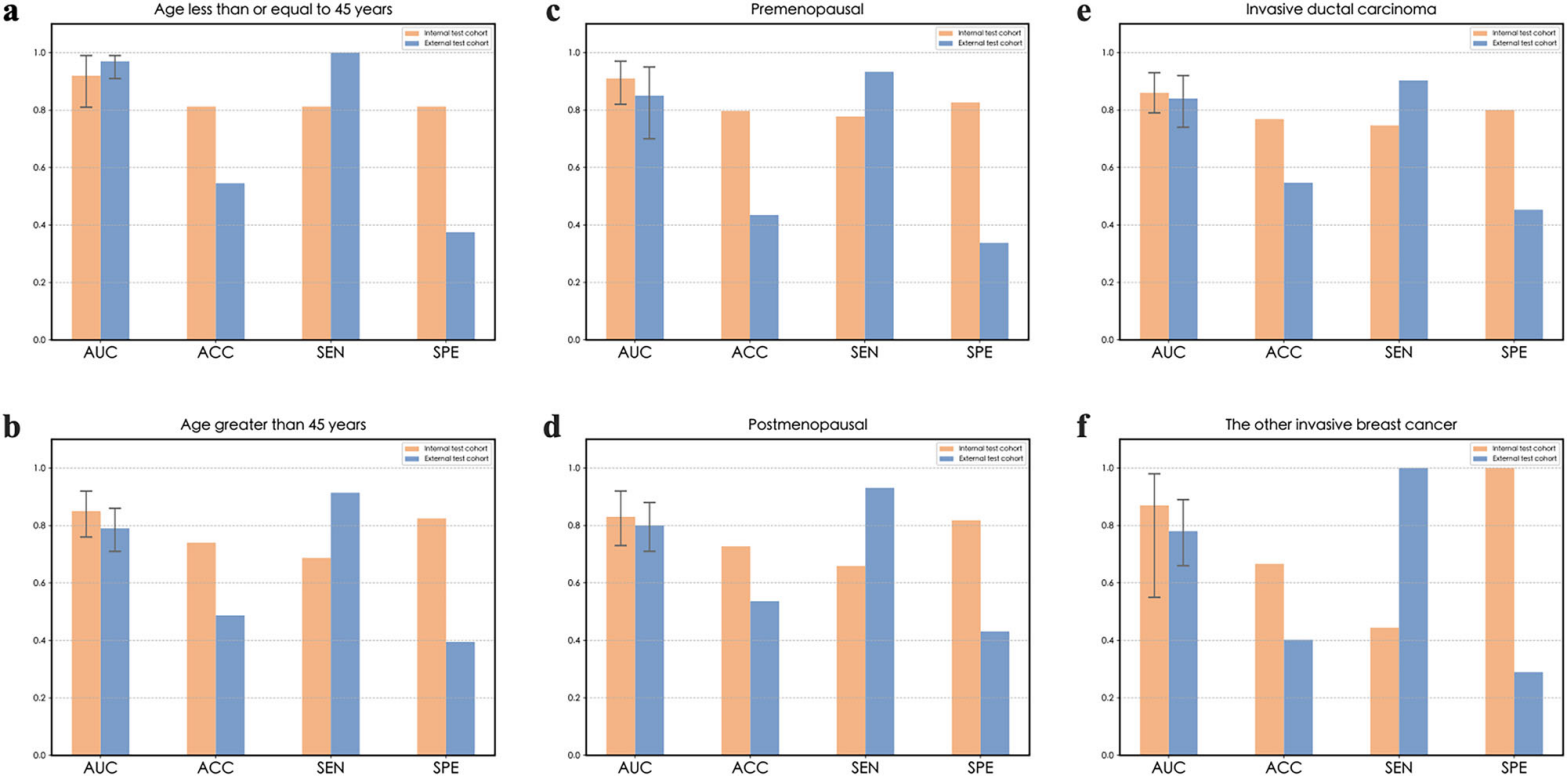
Performance of the fusion model in patient subgroups stratified according to patient age, menstrual status, and pathology. Area under the receiver operating characteristic curve (AUC), accuracy (ACC), sensitivity (SEN) and specificity (SPE) for **a** age less than or equal to 45 years, **b** age greater than 45 years, **c** premenopausal, **d** postmenopausal, **e** invasive ductal carcinoma and **f** the other invasive breast carcinoma in the internal (orange bar) and external test cohorts (blue bar) show that the fusion model achieved high predictive performance in these patient subgroups

Stratification analysis based on age, menopausal status, pathology, lymph nodal status, and tumor size showed that the fusion model maintained high predictive accuracy across all subgroups, with AUCs of 0.83–0.94 in the internal test cohort and 0.78–0.97 in the external test cohort (Figs. 6, S3).

## Discussion

The profound heterogeneity inherent to breast cancer constitutes a key driver of disease progression, with the level of ITH being strongly associated with patient prognosis [19, 20]. The 21-gene RS can reflect the risk of postoperative recurrence in early-stage ER+/HER2− breast cancer and evaluate the benefit of adjuvant chemotherapy. However, this assay is costly, time-consuming, and relatively technically demanding. To address this clinical challenge, this study successfully developed a quantitative indicator named ITHscore, which, our findings suggest, could enable noninvasive, simple, and intuitive characterization of ITH. The ITHscore demonstrated favorable performance in predicting RS (AUC = 0.75–0.76). Furthermore, the addition of ITHscore to the clinical-radiomic model enhanced the fusion model’s predictive performance, yielding AUCs of 0.86 in the internal test set and 0.82 in the external test set.

ITHscore is a novel quantitative parameter that could allow noninvasive and straightforward characterization of ITH. Research on ITHscore remains limited, and to our knowledge, this is the first study to investigate the correlation between ITHscore and RS. In this study, the ITHscore model achieved AUCs of 0.76, 0.75, and 0.75 in the training cohort, internal test cohort, and external test cohort, respectively, demonstrating robust predictive performance. A study by Shi et al [21] published in *Radiology* in 2023 indicated that ITHscore holds potential value in predicting the efficacy of neoadjuvant chemotherapy in breast cancer, with an AUC of 0.74–0.76 in external test datasets. Both that study and ours suggest the potential utility of ITHscore for predicting breast cancer prognosis. Li et al [9] used ITHscore derived from computed tomography images to quantify heterogeneity in non-small cell lung cancer (NSCLC) by integrating local radiomic features and global pixel distribution patterns. Their findings revealed that NSCLC ITHscore serves as an indicator of patient prognosis, which is largely consistent with our results.

Conventional radiomic features have been widely used to characterize ITH and have shown potential value in predicting treatment response and prognosis [22–24]. In this study, the radiomics model achieved an AUC of 0.81 in the training cohort, and AUCs of 0.74 and 0.65 in the internal and external test cohorts, respectively. A study published in 2025 utilized radiomic features from CL and T2W images to develop an RS prediction model, which yielded AUCs of 0.76 and 0.71 in the training and validation cohorts [25], consistent with our findings. The results of this study indicate that the ITHscore model outperformed the radiomics model in both the internal and external test cohorts. This may be because radiomics technology is based on the assumption of a homogeneous mixture within a tumor—an assumption that has been proven incorrect at both tissue and cellular levels [26]. As a result, radiomics tends to overlook local phenotypic differences within tumors. In contrast, habitat analysis segments tumor subregions and quantifies heterogeneity comprehensively from both global and local perspectives, thereby more accurately reflecting lesion structure and function [27, 28].

This study incorporated clinical, conventional radiomic, and ITHscore features to construct multiple RS prediction models. Among them, the fusion model exhibited the highest performance across the training, internal test, and external test cohorts, indicating complementarity among these features. Xu et al [29] found that a model integrating ITH index, traditional radiomic, and clinicopathological features yielded optimal performance in both internal and external validation cohorts for predicting retroperitoneal sarcoma. Similarly, Zhuo et al [30] developed a model combining clinical, imaging, radiomic, and ITH index features to predict the grading of intrahepatic mass-forming cholangiocarcinoma, and their results showed that the combined model performed best. These studies align with our findings, demonstrating that quantitative ITH parameters complement clinical and conventional radiomic features, thereby enhancing the predictive power of models for tumor biological behavior and prognosis. Furthermore, in the feature importance analysis of the fusion model in this study, ITHscore ranked first across all three cohorts, further affirming its critical value in characterizing ITH and predicting RS, as well as its stability and generalizability.

This study has several limitations. First, as a retrospective study, there may be potential bias in population selection, although external validation from a different center was performed to improve reliability. Prospective studies are needed in the future to further investigate the value of quantitative ITH assessment in prognostic prediction. Second, the MRI images were acquired using different scanners with non-uniform imaging parameters. Although signal intensity normalization and uniform spatial resolution were applied, variations in scanning parameters may still increase the instability of the prediction model. Third, while DWI plays an important role in breast cancer management, the Duke dataset did not include DWI sequences, preventing the exploration of its potential value. We look forward to future studies incorporating DWI. Last, despite the promising AUC of our fusion model, we acknowledged a key limitation regarding its clinical applicability, particularly in the external test cohort. The threshold optimized in the training cohort using the Youden index achieved high sensitivity (0.93) but low specificity (0.39) externally, resulting in a high false-positive rate. This discrepancy underscored the challenge of threshold generalizability across heterogeneous populations and the need for recalibration based on clinical context. Future work should focus on prospective validation of thresholds that prioritize clinical utility, such as minimizing false positives for high-stakes decisions.

In conclusion, this exploratory study developed a novel, noninvasive, and intuitive quantitative indicator based on pretreatment breast MRI to characterize intratumoral heterogeneity. This index showed promise of incremental benefit in predicting RS in breast cancer, particularly in clinical settings characterized by limited treatment costs, a need for timely prognostic information, or lack of access to the 21-gene assay. Unlike genomic testing—which typically requires days to weeks and incurs substantial costs—our radiomics and habitat approaches generated rapid, low-cost predictions from routine MRI, offering an accessible alternative in resource-limited environments.

## Supplementary information

ELECTRONIC SUPPLEMENTARY MATERIAL

## Data Availability

The clinical data related to patients and MRI images can be obtained from the corresponding author on reasonable request.

## Abbreviations

AUC: Area under the receiver operating characteristic curve
CL: Last phase of dynamic contrast-enhanced
ER: Estrogen receptor
HER2: Human epidermal growth factor receptor 2
ITH: Intra-tumor heterogeneity
ITHscore: Intratumoral heterogeneity score
ROC: Receiver operating characteristic
RS: Recurrence score
SHAP: Shapley Additive exPlanations
VOI: Volume of interest
