# Supplementary material for: MRI-based quantification of intratumoral heterogeneity for predicting recurrence risk in ER+/HER2− breast cancer
Source: Insights Imaging. 2026 Jun 27;17:177. doi: 10.1186/s13244-026-02342-0 (PMC13310210; doi:10.1186/s13244-026-02342-0)

﻿**MRI-based Quantification of Intratumoral Heterogeneity for Predicting Recurrence Risk in ER+/HER2-**

**Breast Cancer**

**ELECTRONIC SUPPLEMENTARY MATERIAL**

| **Table S1. MRI Protocol** | | | | | | |  |  |
| --- | --- | --- | --- | --- | --- | --- | --- | --- |
| **Datasets** | **Scanner** | **DCE phases** | **Slice thickness (mm)** | **TR/TE (ms)** | **FOV (mm)** | **Matrix** | | **Flip Angle** |
| Institutional dataset | GE 1.5T (Signa HDx) | 5 | 2.8 | 4.8/2.3 | 240×160 | 384×256 | | 15° |
| Institutional dataset | GE 3.0T (Signa HDx) | 5 | 3 | 4.3/2.1 | 225×150 | 384×256 | | 10° |
| Institutional dataset | Siemens 3.0T (Skyra) | 6 | 2.2 | 4.5/1.56 | 360×335 | 384×357 | | 10° |
| Institutional dataset | ﻿Aurora 1.5T (﻿Dedicated Breast MRI Systems) | 4 | 1.5 | 29/4.8 | 360×360 | 360×360 | | 90° |
| Duke dataset | GE 1.5T (Optima MR450w) | 5 | 1.1~2.2 | 5.2-6.7/2.4-2.7 | 280-380 | 360×360 | | 10° |
| Duke dataset | GE 3T (SIGNA EXCITE) | 5 | 1.6 | 6.2/2.5 | 300 | 350×350 | | 12° |
| Duke dataset | GE 1.5T (SIGNA HDx) | 5 | 2 | 4.9-5.0/2.3-2.4 | 300-370 | 340×340 | | 10° |
| Duke dataset | GE 3T (SIGNA HDx) | 5 | 1.1-2 | 5.1-6.7/2.4-2.6 | 290-390 | 350×350 | | 10° |
| Duke dataset | GE 1.5T (Signa HDxt) | 5 | 1.1-2.2 | 4.8-6.3/2.2-2.7 | 300-390 | 340×340/384×360 | | 10° |
| Duke dataset | GE 3T (Signa HDxt) | 5 | 1.1-2.0 | 5.1-7.1/2.4-2.8 | 280-400 | 350×350/384×384 | | 10° |
| Duke dataset | Siemens 1.5T (Avanto) | 5 | 1.1-2.0 | 4.1-4.4/1.3-1.6 | 300-400 | 320×320/448×448 | | 10°/12° |
| Duke dataset | Siemens 3T (Skyra) | 5 | 1.1-2.0 | 3.8/1.4 | 346-400 | 448×381 | | 10° |
| Duke dataset | Siemens 2.9T (Trio) | 5 | 1.1 | 3.5/1.3 | - | 448×448 | | 10° |
| Duke dataset | Siemens 3T (TrioTim) | 5 | 1.1-2.5 | 3.7-4.1/1.4-1.6 | 300-380 | 448×448 | | 10°/7° |
| *TR* time to repetition, *TE* time to echo, *FOV* field of view, *DCE* dynamic contrast enhancement. | | | | | | | | |

| **Table S2. Features in different models** | | |
| --- | --- | --- |
| **Model** | **Number of features** | **Features** |
| **Fusion** | 6 | ITHscore Original_shape_Sphericity Original_glszm_ZoneVariance Wavelet-LLL_firstorder_Skewness Log-sigma-3-0-mm-3D_firstorder_90Percentile Invasive malignancy grade |
| **Clinical-radiomics** | 8 | Wavelet-LLH_firstorder_Kurtosis Wavelet-LLL_firstorder_Skewness Wavelet-LLL_glrlm_LongRunLowGrayLevelEmphasis Log-sigma-2-0-mm-3D_glcm_ClusterProminence Log-sigma-3-0-mm-3D_gldm_LargeDependenceHighGrayLevelEmphasis Progesterone receptor status, Lymph node status, Invasive malignancy grade |
| **ITHscore** | 1 | ITHscore |
| **Radiomics** | 3 | Wavelet-LHH_gldm_SmallDependenceLowGrayLevelEmphasis Wavelet-LLL_firstorder_Skewness Log-sigma-3-0-mm-3D_firstorder_90Percentile |
| **Clinical** | 5 | Progesterone receptor status, Lymph node status, Tumor size, Pathology, Invasive malignancy grade |
| *ITHscore* intratumoral heterogeneity score, *glszm* gray-level size zone matrix, *glrlm* gray level run length matrix,  *glcm* gray-level co-occurrence matrix, *gldm* gray-level dependence matrix. | | |

﻿Figure S1: Intratumoral heterogeneity score (ITHscore) distribution bar chart of low and high recurrence risk. ITHscores were higher in the high-recurrence risk than the low-recurrence risk in the training, internal test, and external test cohorts (p < 0.001).


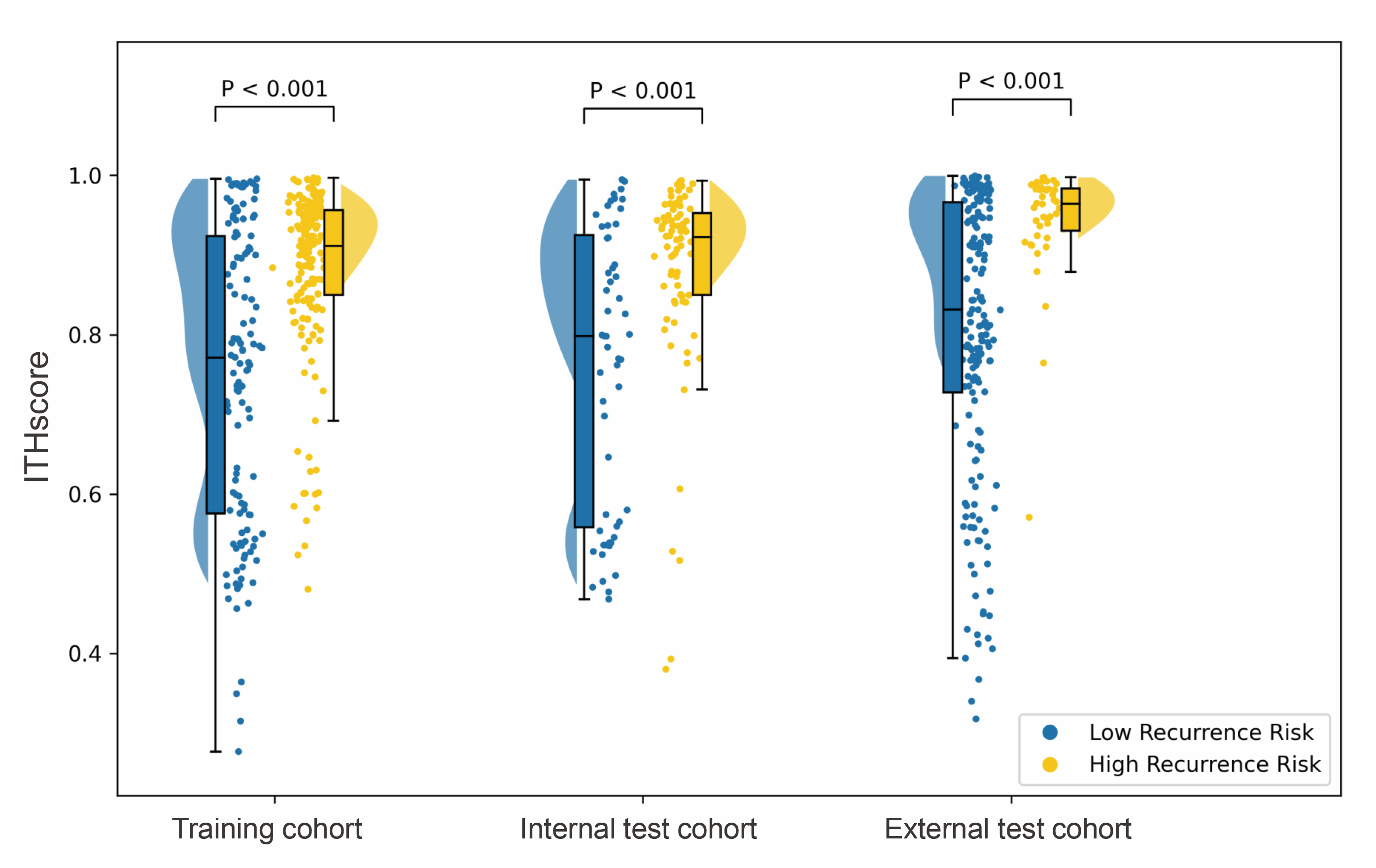


Figure S2: Pearson correlation coefficients in the fusion model.


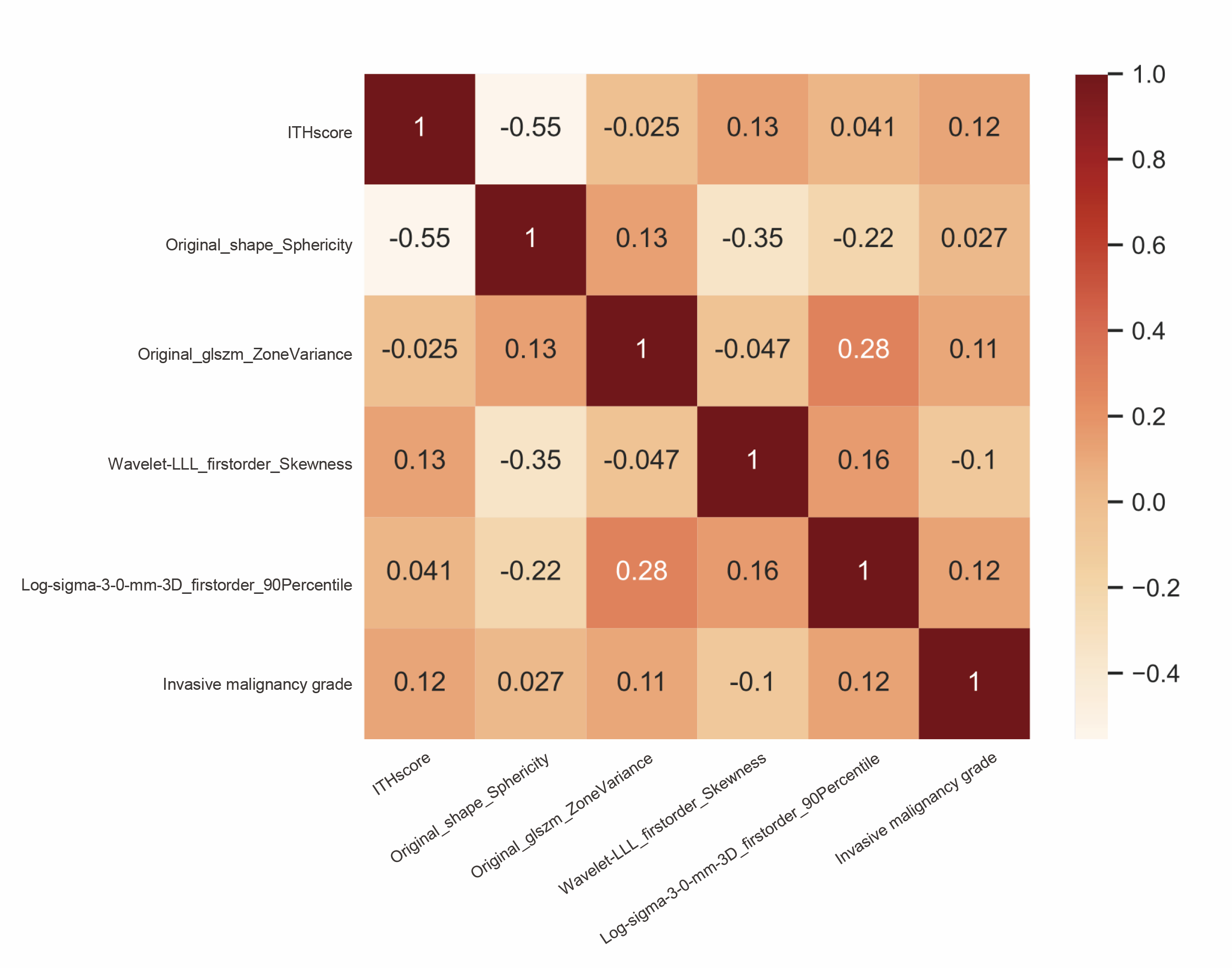


﻿Figure S3: Performance of the fusion model in patient subgroups stratified according to lymph node status and tumor size. Area under the receiver operating characteristic curve (AUC), accuracy (ACC), sensitivity (SEN) and specificity (SPE) for (a) negative lymph node, (b) positive lymph node, (c) pTII/III and (d) pTI in the internal (orange bar) and external test cohorts (blue bar) show that the fusion model achieved high predictive accuracy in patient subgroups.


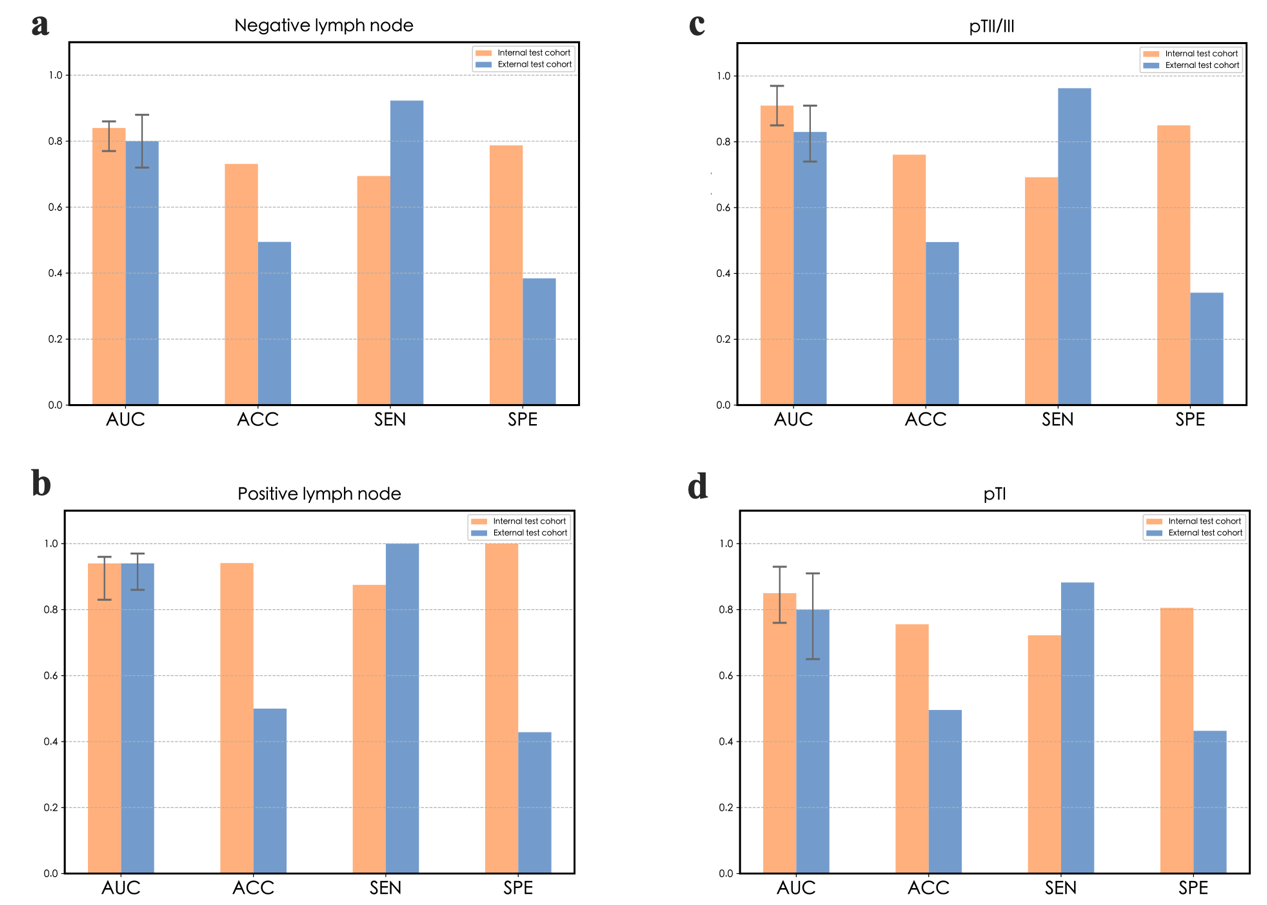


﻿Figure S4: Calibration curves of the fusion model in the internal (a) and external (b) test cohorts.


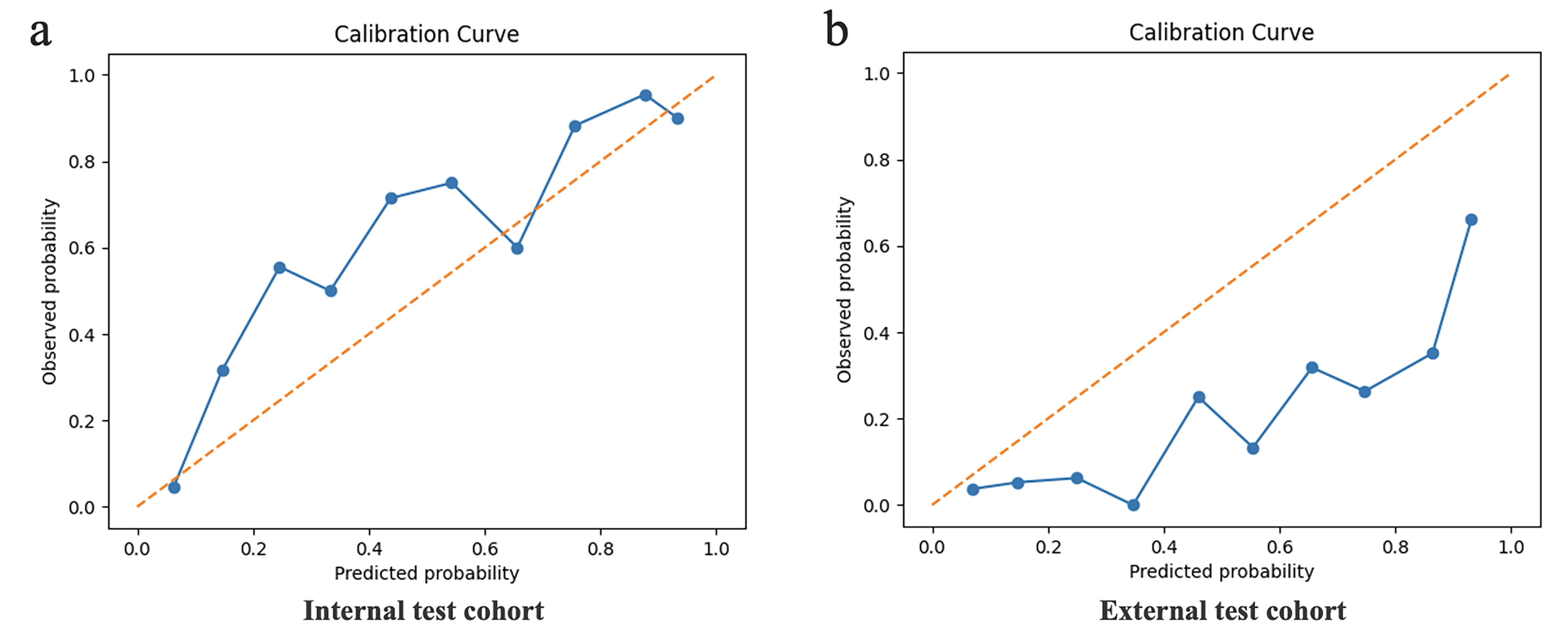

Supplement: Supplementary file 1 — ELECTRONIC SUPPLEMENTARY MATERIAL [file 13244_2026_2342_MOESM1_ESM.docx]
